# Dissecting the Genetics of Autism Spectrum Disorders: A *Drosophila* Perspective

**DOI:** 10.3389/fphys.2019.00987

**Published:** 2019-08-07

**Authors:** Paola Bellosta, Alessia Soldano

**Affiliations:** ^1^Laboratory of Metabolism of Cell Growth and Neuronal Survival, Department of Cellular, Computational and Integrative Biology (CIBio), University of Trento, Trento, Italy; ^2^Department of Medicine, New York University Langone Medical Center, New York, NY, United States; ^3^Laboratory of Translational Genomics, Department of Cellular, Computational and Integrative Biology (CIBio), University of Trento, Trento, Italy

**Keywords:** autism (ASD), shank, FMR1, neurexin, neuroligins, mGlu receptor 5, *Drosophila*, dopamine

## Abstract

Autism Spectrum Disorder (ASD) is a complex group of multi-factorial developmental disorders that leads to communication and behavioral defects. Genetic alterations have been identified in around 20% of ASD patients and the use of genetic models, such as *Drosophila melanogaster*, has been of paramount importance in deciphering the significance of these alterations. In fact, many of the ASD associated genes, such as *FMR1, Neurexin, Neuroligins and SHANK* encode for proteins that have conserved functions in neurons and during synapse development, both in humans and in the fruit fly. *Drosophila* is a prominent model in neuroscience due to the conserved genetic networks that control neurodevelopmental processes and to the ease of manipulating its genetics. In the present review we will describe recent advances in the field of ASD with a particular focus on the characterization of genes where the use of *Drosophila* has been fundamental to better understand their function.

## Autism

Autism Spectrum Disorder (ASD) is a complex developmental neurological disease characterized by persistent deficits in social behaviors (communication, interaction), presence of repetitive and restrictive comportments and is often associated with motor deficits and sleep abnormalities, among others. Among individuals suffering from ASD, there is a high frequency of intellectual disability and mental retardation, although the described frequency is variable due to the difficulty in assessing cognitive performance in certain groups of ASD patients (O’Brien and Pearson, 2004; Chakrabarti and Fombonne, 2005). Autism is not considered a single gene disorder because it is caused by both genetic and non-genetic risk factors that induce a complex range of different symptoms for which the precise causes are unknown (Park et al., 2016). Genetic disorders, such as Fragile X syndrome (FXS), Down syndrome, and, more recently, Asperger’s and Rett syndrome, have been associated with ASD. In less than 20% of patients has a clear monogenic cause for ASD been identified and most of these studies highlighted mutations in genes involved in several aspects of synapse biology, such as synaptogenesis/synaptic plasticity/morphology/function and axon motility (De Rubeis et al., 2014; Iossifov et al., 2014; Luo et al., 2018).

## Asd Associated Defects in Synaptogenesis and Synaptic Plasticity

The identification of ASD susceptibility genes involved in various aspects of synapse biology, lead to the hypothesis that aberrant synaptogenesis/synaptic function might be a central process in ASD (Peca and Feng, 2012; Zoghbi and Bear, 2012). Multiple studies in animal models converge on the concept that reproducing alterations in ASD genes leads to aberrant synaptic morphology and function (Peca and Feng, 2012; Zoghbi and Bear, 2012). Interestingly, observation of post-mortem ASD patients’ tissues indicate that dendritic spines, postsynaptic sites in the mammalian brain, are present at a higher density in ASD patients and this condition is most commonly found in ASD subjects with lower levels of cognitive performance (Hutsler and Zhang, 2010). Moreover, ASD patients have an increased density of dendritic spines in layer V pyramidal neurons and reduced developmental spine pruning, a process needed to achieve correct neuronal communication (Tang et al., 2014). This is of particular interest since it has been postulated that ASD might be caused by an altered balance between excitatory and inhibitory synapses, probably due to defects in synapse elimination/formation (Ramocki and Zoghbi, 2008; Gatto and Broadie, 2010).

### *Drosophila* as a Model to Study ASD

*Drosophila* is an excellent model to study ASD to understand the consequences of genetic alterations found in ASD patients and to identify the molecular mechanisms underlying the role of ASD related genes in synaptic function and plasticity (Doi et al., 2016; Tian et al., 2017). Moreover, 75% of the human disease genes have orthologs in *Drosophila* (Bier, 2005), rendering the fruit fly a highly tractable genetic model organism to understand the molecular bases of ASDs. In the past decade the panel of genetic tools that can be used to study human disease genes has expanded massively (Table 1; Chow and Reiter, 2017). *Drosophila* has been used for classical **unbiased screens**, using either mutagens to induce random mutations in the genome or genome-wide RNAi/CRISPR screens, to identify genes that lead to ASDs-like phenotypes. On the other hand, known ASDs genes have been perturbed to mimic the patient’s condition and to study the biological consequences of these alterations.

**TABLE 1:** Summary of the genetic tools that can be used to study the physiological role of ASD genes and to understand their contribution, alone and in combination with others, to ASD development.

**Genetic tool**	**Application to ASDs**
Binary system such as Gal4/UAS system, LexA/LexAop, Q-system	– Overexpression or silencing of ASD associated genes to mimic deletions or amplifications in patients. – Overexpression of ASD genes harboring mutations found in patients in a knockout background.
CRISPR genome engineering	– Engineering of the *Drosophila* genome to induce, when possible, genetic alterations similar to the ones observed in ASD patients. – Engineering of the *Drosophila* genome to induce the knockout or overexpression of ASD genes. – Creation of “patient specific” *Drosophila* models where the endogenous gene is replaced with the patient variant.
GeneSwitch Gal4 system (GS)	– Tissue and time specific overexpression or silencing of ASD associated genes to mimic deletions or amplifications in patients –Overexpression of ASD genes harboring mutations found in patients in a knockout background.
Clonal analysis system: MARCM, QMARCM, twin-spot MARCM.	– Overexpression or silencing of ASD genes in a subset of cells in an otherwise wt tissue to understand the contribution of the overexpressed genes to the tissue’s functionality and development. The same experiment can be performed in other mutant backgrounds. – Overexpression of disease variant human ASD genes to understand the contribution of the mutations to the tissue’s functionality and development.

**FIGURE 1:**
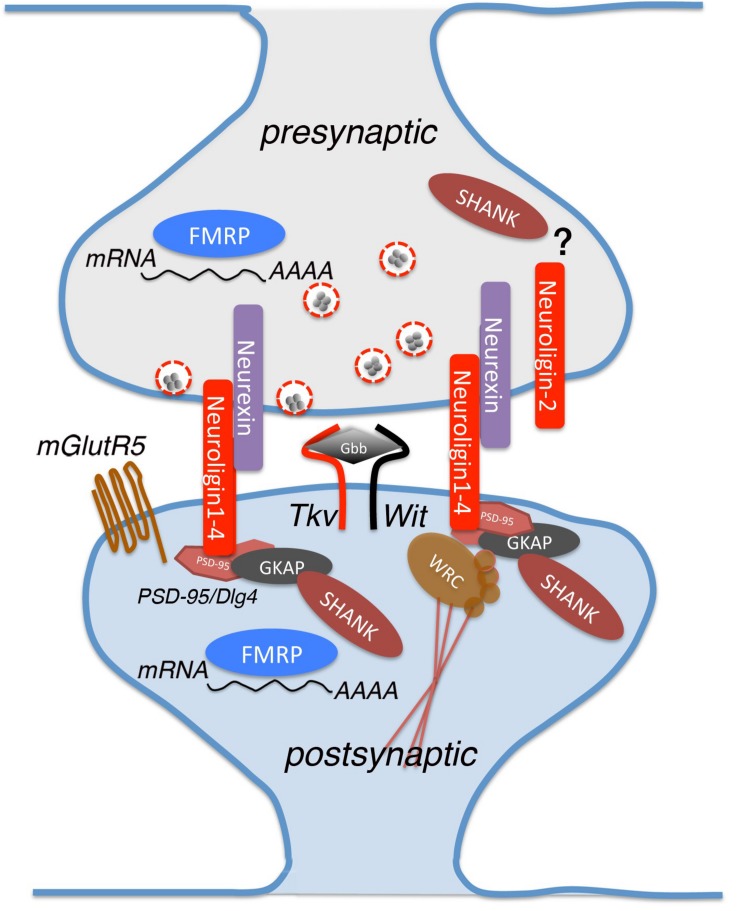
A schematic view of a glutamatergic synapse, showing the proteins analyzed in this review and implicated in Autism Spectrum Disorder (ASD). Neurexin and neuroligin are located in pre- and post-synaptic sites where they interact with multiple partners for mediating synapse development and maturation. Neuroligins binds to PSD-95, which interacts indirectly with Shank by intracellular complexes containing also Guanylate-kinase-associated protein (GKAP). dNlg1 and 3 act mostly in pre-synaptic terminals while dNlg2 functions is both pre- and post-synaptic ends. Other proteins involved in the synaptic establishment are FMRP RNA-binding and Thickveins (Tkv), that are expressed in both synaptic sites, mGlutR5 and Wishful thinking (Wit) that are found specifically on the post-synaptic membrane. Glass Bottom Boat (Gbb) is the ligand of both Tkv and Wit, homologous to vertebrate bone morphogenic protein (BMP).

In the present review we describe the latest studies that use *Drosophila* to clarify the function of the most representative genes associated with ASD (Figure 1).

### *dfmr*1

Fragile X syndrome (FXS) is a neuro-developmental disease that leads to intellectual disability and is the most common form of autism of monogenic origin (Mila et al., 2018). FXS is caused by a variable expansion of a trinucleotide (CGG) repeat in the 5′ UTR of the *fragile X mental retardation-1* gene (*FMR1*), or less frequently, by point mutations in *FMR1* (Collins et al., 2010; Handt et al., 2014), that leads to loss of FMR1 protein (Pieretti et al., 1991). *FMR1* encodes for an RNA-binding protein, FMRP, that mainly inhibits translation by binding to specific sequences on mRNAs (Darnell et al., 2011; Ascano et al., 2012).

*Drosophila* harbors only one *FMRP* ortholog, *dfmr1*, that shares high homology with its mammalian counterpart (Wan et al., 2000). A recent study using fruit flies suggested that the **molecular function** of *dfmr1* might not only be translation repression. Ribosome-profiling of oocytes upon *dfmr1* knockdown shows that *dfmr1 RNAi* leads to both enhanced and reduced mRNA translation in proportion to protein size, with dmfr1 predominantly up-regulating bigger proteins (Greenblatt and Spradling, 2018). Interestingly, many of the down-regulated genes are orthologs of genes implicated in ASD, such as the E2 Ubiquitin-conjugating enzyme BIRC6, or the Vacuolar H + ATPase DMXL2, both of which are associated with intellectual disabilities and neurodevelopmental disorders in humans, a result that outlines the relevance of using *Drosophila* genetics to gain insights into these human pathologies.

*dfmr1* plays a central role in **synaptic plasticity,** indeed loss-of-function mutants of *dfmr1* show synaptic overgrowth, increased number and enlargement of synaptic boutons, and excessive branching at the Neuromuscular Junctions (NMJ). Mutations in *dfmr1* affect synaptic transmission at histaminergic photoreceptor synapses (central) and glutamatergic NMJ synapses (peripheral) (Zhang et al., 2001).

*dfmr1* controls **brain development and neural circuit assembly** (Morales et al., 2002). Loss of *dfmr1* causes axon extension defects of **Dorsal Cluster neurons (DC)** and **lateral neurons (LNvs)**, and neurite-branching abnormalities in DC neurons. Interestingly, loss and gain of function of *dfmr1* lead to similar phenotypic defects, indicating that the levels of *dfmr1* are critical for brain development (Morales et al., 2002). The role of *dfmr1* in regulating axon morphology has also been demonstrated in the **Mushroom Body neurons (MB)**, a higher hierarchy circuit involved in olfactory learning and memory (Lee et al., 1999; Akalal et al., 2006). Loss of *dfmr1* in all MB neuronal classes increases structural complexity and induces growth of additional processes from neuronal soma, supporting the overbranching and overgrowth phenotype visible in dendrites and axons (Michel et al., 2004; Pan et al., 2004; Tessier and Broadie, 2008). *dfmr1* also controls remodeling of two classes of MB body extrinsic input and output neurons, namely GABA-ergic MVP2 (MBON-γ1pedc > α/β) and projection neuron (PN). In fact, the dendritic arborizations of these neurons are enlarged in *dfmr1* null animals. MVP2 and PN neurons respond in the opposite way to this activity by remodeling their dendritic arbor and *dfmr1* is required for this function (Doll and Broadie, 2015).

Loss of *dfmr1* causes several **behavioral defects** including deficits in **memory** (Coffee et al., 2010; Gatto et al., 2014), associative **learning** defects (Choi et al., 2010; Santos et al., 2014; Doll and Broadie, 2016) and alteration of **circadian rhythm** (Sofola et al., 2008; Gatto and Broadie, 2009), which are all known to be linked to defects in LNvs morphology (Dockendorff et al., 2002; Sofola et al., 2008). Gene expression analysis at different times during the day (Circadian-time points CT) highlighted a subset of mRNAs and miRNAs that, in *dfmr1* mutant flies, were altered at a specific time point only. This pattern of gene expression alteration reflects a circadian rhythm-dependent alteration (Xu et al., 2012). *dfmr1* mutants also exhibit **sleep defects:** showing a prolonged “sleep phase”, which is reduced by overexpression of *dfmr1* in the MB (Bushey et al., 2009), and a deeper sleep (night-like) phenotype at daylight (van Alphen et al., 2013). Similarly, patients with FXS suffer from sleep disorders suggesting a conserved function of *FMR1* in controlling components of the circadian rhythm.

*dfmr1* modulates **grooming behavior**, recapitulating the repetitive behavior observed in ASD patients, and is therefore of great relevance for translational studies. *dfmr1* mutants groom more than control flies; this phenotype worsens with age and can be suppressed by treatment with reserpine, which blocks the *Drosophila* vesicular monoamine transporter (dVMAT) (Tauber et al., 2011).

*dfmr1* mutants show also impairment in **odor-induced attraction and aversion**, due to reduced lateral interactions across the olfactory glomeruli and impairment of the lateral inhibition in the antennal lobe caused by weaker inhibition from GABAergic interneurons (Franco et al., 2017). These results are of great interest in comparative studies in humans given that alterations in GABAergic transmission and lack of inhibition might be central components of the neuropathology of FXS.

FXS patients, together with most individuals with ASD, suffer from **dysfunctions in sensory processing (SPD)**, meaning they respond to a certain behavioral stimulus differently than individuals in the average population (Sinclair et al., 2017). This dysfunction has been investigated in flies by studying the sensory processing of the *Drosophila* stress odorant (dSO) (Androschuk et al., 2018). *dfmr1* null animals have lost dSO avoidance-behavior and *dfmr1* is required in the MB and glia to mediate the dSO sensory response. This behavioral defect can be pharmacologically rescued by feeding adults with molecules that target cAMP/cGMP signaling pathways, such as the cAMP-increasing agent IBMX (3-isobutyl-1-methylxanthine), and the cAMP-dependent PKA activator and the cGMP dependent phosphodiesterase inhibitor 8-CPT (8-(4-Chlorophenylthio)adenosine 3′,5′-cyclic monophosphate), suggesting a potential use of these drugs In ASD treatments (Franco et al., 2017).

Up to now only few potentially **pathogenic mutations** have been identified in FXS patients. The most studied is an isoleucine to asparagine substitution (I304N) within the second K-homologous (KH) domain of the human FMRP, which is associated with very severe FXS (De Boulle et al., 1993). However, mutations in the highly conserved isoleucine residues I244N and I307N of the KH domain in *Drosophila* resulted in *dfmr1* null-like, MB β-lobe midline crossing phenotype, though at a lower frequencies than in *dfmr1* mutants. These KH mutants also fail to retain rhythmic locomotion activity in constant darkness, but with a milder phenotype than in *dfmr1* null animals (Banerjee et al., 2007). More recently, Okray et al. (2015) characterized in *Drosophila* a new FMR1 frameshift mutation (Guanine insertion in *exon-15*) found in a patient with FXS. This mutation generates a novel peptide sequence with a premature stop codon, resulting in the truncation of the FMRP protein at the C-terminus and loss of the arginine-glycine-rich motif (RGG box), which is one of the FMRP RNA-binding domains. Overexpression of a mutant form of *dfmr1 (dfmr1-^Δ*C+NLS*^* allele), which closely mimics the human variant, in LNvs, results in axons that fail to extend medially, leads to aberrant bifurcations of axonal bundle and to the formation of axonal “tangles.”

### Neurexin and Neuroligins

Neurexin (Nrx) and Neuroligins (Nlgs) are adhesion molecules that function as *trans*-synaptic binding partners involved in synaptogenesis (Knight et al., 2011). Several genetic alterations including point mutations, deletions and translocation events have been identified in *NRXN1*, *NLGN3* and *NLGN4* in ASD patients (Laumonnier et al., 2004; Kim et al., 2008; Yan et al., 2008).

In *Drosophila*, loss of *dNlgs* (*Drosophila* harbors 4 *dNlgs*) and *dNrx* results in developmental defects at the NMJ such as an altered number of boutons, aberrant **presynaptic/postsynaptic** structure, and impaired synaptic transmission (Sun et al., 2011; Chen et al., 2012; Hahn et al., 2013; Xing et al., 2014). In particular, dNlg1, 2, and 4 have a positive effect on synaptic growth at the NMJ and their loss leads to a reduction of synaptic boutons (Banovic et al., 2010; Sun et al., 2011), while loss of dNlg3 leads to the opposite phenotype (Xing et al., 2014). dNlg1 and 3 act mostly in pre-synaptic terminals while dNlg2 functions in both pre- and post-synaptic ends (Chen et al., 2012; Xing et al., 2014). dNlgs and dNrx work together to coordinate these functions.

Recent studies dissected the **molecular mechanisms** underlying these functions: Zhang and colleagues (Zhang et al., 2017) demonstrated that dNlg4 modulates BMP signaling by maintaining the protein levels of the type-I BMP receptor Thick Veins (Tkv) at the presynaptic sites. **BMP signaling** seems to be a target of several dNlgs/dNrx complexes; in fact, it has been demonstrated that Tkv levels are also reduced in dNlg1 and dNrx mutants (Banerjee et al., 2017). Interestingly, mutants of the type-II BMP receptor Wishful Thinking (Wit) show phenotypic similarities to dNlg1 and dNrx mutants (Banerjee and Riordan, 2018). dNrx, dNlg1 and Wit seem to form a complex at the NMJ, where dNrx and dNlg1 are required for both localization and stability of Wit. dNrx is found in a complex with Wit and its ligand Gbb, the ortholog of vertebrate BMP, and other downstream effectors to allow proper axonal transport and microtubule organization (Banerjee and Riordan, 2018).

dNlg1 also directly affects the actin cytoskeleton via interaction with the WAVE regulatory complex (WRC), one of the key players in **F-actin assembly** (Xing et al., 2018). In particular, dNlg1 mediates the effect of dNrx on actin at post-synaptic terminals by binding to the WRC and recruiting it to the post-synaptic membrane. dNlg1-WRC interaction mediates post-synaptic F-actin assembly, which is required for normal NMJ assembly and boutons growth, while dNrx and dNlg4 control axonal branching (Liu et al., 2017). dNrx is also expressed in the axon terminals and interstitial branches of L4 lamina neurons that project into the medulla neuropil, and is required for L4 columnar restriction. In particular, dNlg4/dNrx interaction promotes dNrx clustering on the membrane which results in dNrx/Ephrin interaction and subsequent Ephrin clustering (Neriec and Desplan, 2016).

In mammals, Neurexins and Neuroligins are also central for the establishment of functional synaptic networks (Sudhof, 2017). The findings described in *Drosophila* strongly support that dNlgs and dNrx have a primary role in synapse formation/maintenance and outline how these signaling pathways might be further assessed as pharmacological targets.

### Shank

The family of SH3 and multiple ankyrin repeat domains proteins (SHANKs) is composed of three members: SHANK1, 2 and 3. These proteins are scaffolding proteins present at the post-synaptic density in glutamatergic synapses. SHANK3 deletions, duplications, and mutations have been frequently reported in patients with ASD (Durand et al., 2007; Boccuto et al., 2013; Leblond et al., 2014). *SHANK3* mutations are one of the most prevalent monogenic causes of ASD, accounting for at least 0.69% of all cases, and patients harboring SHANK3 truncating mutations display autism combined with moderate to severe intellectual disabilities. Moreover, 22q13.3 deletion syndrome, also known as Phelan–McDermid syndrome, which is characterized by ASD or ASD-traits, is caused by deletions and mutations that lead to the loss of a functional copy of *SHANK3* (Soorya et al., 2013). Recent META-analysis of SHANK family mutations in ASD identified deletions disrupting *SHANK1* and *SHANK2* genes in patients, but not duplication of either (Leblond et al., 2014). This study also suggested the existence of a gradient of severity in **cognitive impairment** depending on the SHANK gene mutated. So far, the molecular mechanisms underlying SHANK functions remain partially unclear and studies using *Drosophila* have contributed significantly in addressing this question.

*Drosophila* harbors only one ortholog of the SHANK family called Prosap/Shank (Liebl and Featherstone, 2008). Harris et al. (2016) described that Shank localizes to the post-synaptic membrane at the NMJ where it is involved in the regulation of synapse morphology and maturation. The levels of Shank at **synapses** are critical; *Shank* mutants exhibit a 24% reduction in synaptic boutons and an excessively high number of immature synaptic structures. On the other hand, *Shank* heterozygous animals show an intermediate phenotype, with a 15% reduction in boutons numbers but no increase in immature synaptic structures. Interestingly, post-synaptic Shank overexpression leads to phenotypes similar to those observed in *Shank* mutants, confirming that the levels of Shank are critical to achieve normal synaptic development. Shank defects have been associated with the modulation of Wnt/FNI (Frizzled Nuclear Import) pathway at the post-synaptic terminal. Shank affects the internalization of the Frizzled-2 (Fz2) receptor, most likely by organizing molecules associated with its internalization and trafficking to the nucleus (Harris et al., 2016). A more recent study from Wu et al. (2017) described Shank expression in axons and at the presynaptic terminal, but not at the post-synaptic sites of the NMJs. Moreover, they generated new *Shank* mutant alleles that show normal morphology at the NMJ and at the post-synaptic density. The authors focused on the role of Shank in the CNS since the protein, like its mammalian counterpart, is expressed in the brain and enriched in the neuropil region. Loss of *Shank* leads to developmental defects of the synapses in the larval MB Calyx, where the protein exerts its function at both pre- and post-synaptic sites. Synapse defects are visible also in the adult MB Calyx, that presents altered microglomeruli and abnormal localization of the α7 subunit of nicotinic acetylcholine receptor (AChR Dα7) and Choline acetyltransferase (ChAT). These abnormalities result in significant impairment of the olfactory learning in *Shank* mutants.

### mGluR

A genome-wide association study (GWAS) of copy-number variation (CNVs) in patients with autism that lead to defective gene family interaction networks (GFINs) (Hadley et al., 2014) identified CNVs in the metabotropic glutamate receptor (mGluR) signaling pathway in 5.8% of patients with ASD.

The involvement of mGluR in autism has been highlighted in its involvement in FXS. The “**mGluR theory**” states that loss of *FMRP* in FXS results in increased glutamatergic signaling via mGluR5, leading to uncontrolled increases in local mRNA translation (Pop et al., 2014). In fact, mGluR activation normally stimulates synthesis of proteins involved in stabilization of long-term depression (LTD) (Weiler et al., 1997). In FXR patients, this translation stimulation is not balanced by the presence of FMRP and leads to increased AMPA receptor internalization and destabilization of the synapses.

As described in the previous section, loss of *dfmr1* activity in *Drosophila* mimics classic FXS symptoms and the impact of **mGluR inhibition** on these phenotypes has been studied by several groups. McBride et al. (2005) demonstrated that **treatment with mGluR antagonists** or Lithium Chloride (LiCl), during development and adulthood, restores the naive courtship levels of the *dfmr1* mutants. Similar treatments also rescue *dfmr1* defects in immediate recall-memory and the lack of short-term memory. Moreover, the treatment with mGlur antagonists greatly reduces axon growth defects (β lobe overgrowth) observed in the MB of *dfmr1* mutants. Interestingly, the free running rest-activity rhythm defects of *dfmr1* mutant flies are not rescued by these treatments, suggesting that not all the phenotypes observed in *dfmr1* null flies are due to upregulation of mGluR signaling (McBride et al., 2005). Recently, the study of the relationship between mGluR and *dfmr1* was extended by investigating the effect of aging on *dfmr1* mutants. In particular, Choi et al. (2010) demonstrated that *dfmr1* mutants show an age-dependent loss of learning that was rescued by the administration of mGluR antagonists and LiCl. Interestingly, treatment during development rescued the learning defect but not the courtship phenotype, indicating that the rescue obtained by treatment during development alone is not permanent. In fact, when aged flies were treated during development and adulthood or during adulthood alone, the naive courtship was restored (Choi et al., 2010). The interconnection between *dfmr1* and mGluR has been demonstrated also through **genetic interaction,** where loss of *dfmr1* was shown to partially alleviate the phenotypes at the NMJ resulting from loss of *mGluR*, possibly via reduction of translational inhibition. Similarly, loss of *mGluR* partially rescues the defects caused by loss of *dfmr1* and the consequent impairment of translation regulation (Repicky and Broadie, 2009).

### Dopamine Network

The dopamine (DA) network has been widely associated with ASD, where mutations in genes of the DA signaling, such as the Dopamine transporter (DAT), Synataxin 1 (STX1), the DA-receptors, and enzymes involved in DA metabolism, have been associated with autism. Work from several groups suggested that dopamine imbalances in specific circuits of the brain could lead to ASD related behavior (Gadow et al., 2010; Nakamura et al., 2010; Paval, 2017). Moreover, increased size of DA-enriched brain regions, such as the striatum, has been associated with the severity of the disorder (Langen et al., 2014).

Several years ago, a new missense mutation in the human *DAT* gene (**hDAT-T356M**) was identified. This mutation results in reduced ability to accumulate intracellular DA, due to an increased dopamine efflux (Hamilton et al., 2013). The functional consequences of this mutation have been studied in *Drosophila* by expressing the hDAT-T356M in *DAT* null mutant flies. These animals show hyperactivity as compared to flies expressing the wt *hDAT* gene due to increased extracellular levels of DA and abnormal dopamine efflux (Hamilton et al., 2013).

Exome sequencing studies in ASD patients led to the identification of missense variants in the *hDAT* (**hDAT-R51W**) and in *STX1A* (**STX1A-R26Q**) genes. The analysis of these mutations showed defects in the reverse transport of DA that leads to behavioral abnormalities (De Rubeis et al., 2014; Iossifov et al., 2014; Cartier et al., 2015). Mechanistically, the STX1A-R26Q variant is less phosphorylated by Caseine Kinase-2 (CK2), a modification that supports the reverse transport of DA and leads to a reduction in DA efflux. Similarly, the hDAT-R51W variant shows a reduced interaction with STX1 and reduced DA efflux. The effects of these mutations have been characterized *in vivo* in *Drosophila* by assessing locomotion. In fact, Amphetamine (AMPH) feeding stimulates *Drosophila* locomotion but only in the presence of a fully functional DA network. Moreover, expression of a dominant negative form of CK2, mimicking the STX1A-R26Q variant, in DA neurons renders flies insensitive to AMPH. On the other hand, flies harboring the hDAT-R51W mutation increased their locomotion upon AMPH significantly less than wt hDAT expressing flies, confirming the reduced ability of AMPH to cause DA efflux in hDAT R/W mutants (Cartier et al., 2015).

## Concluding Remarks

**FIGURE 2:**
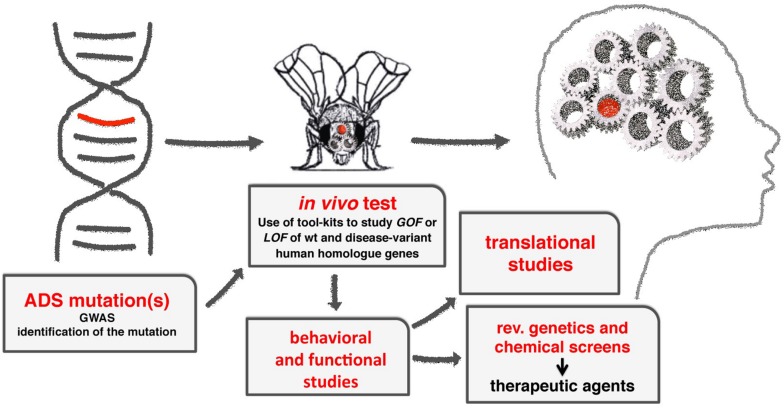
A diagram showing the flow-through to study the molecular mechanisms of ADS using Drosophila. From the initial identification of human genes associated to ADS, to the characterization of their functions using GOF and LOF experiments, applying the milieu of genetic tools available (see Table 1). Using transgenic animals, functional and behavioral studies are used to provide the translational benefits necessary to identify or clarify the function of the gene in humans. Moreover, the fruit fly can be easily adapted to perform reverse genetics or chemical screens to identify novel genes or therapeutic drugs in ADSs.

*Drosophila melanogaster* is an extremely useful model to understand the molecular mechanisms underlying the function of ASD associated genes in brain development and function (Figure 2).

Moreover, the fast growing body of GWAS provides detailed information on the presence of genomic alterations in patients, for which the functional consequences and their relevance in ASD are difficult to interpret (i.e., gene redundancy, complex networks etc.). The fruit fly allows for the analysis of the effects of multiple genetic modifications in different subsets of cells, allowing for the discrimination of the contributions of combinations of genetic alterations co-occurring in ASD patients.

Therefore, the combination of genomic analysis of ASD patients together with the use of an easy to manipulate *in vivo* model with a robust and comparable neuronal development, will be essential to gain insight into the pathogenesis of these disorders.

## Author Contributions

Both authors listed contributed to writing and reviewing the manuscript.

## Conflict of Interest Statement

The authors declare that the research was conducted in the absence of any commercial or financial relationships that could be construed as a potential conflict of interest.
